# Discovery of under immunized spatial clusters using network scan statistics

**DOI:** 10.1186/s12911-018-0706-7

**Published:** 2019-02-04

**Authors:** Jose Cadena, David Falcone, Achla Marathe, Anil Vullikanti

**Affiliations:** 1 Biocomplexity Institute & Initiative, University of Virginia, 995 Research Park Boulevard, Charlottesville, VA 22911 USA; 2 Department of Computer Science, University of Virginia, Charlottesville, VA 22911 USA; 3 Department of Public Health Science, University of Virginia, School of Medicine, Charlottesville, VA 22911 USA; 4 Lawrence Livermore National Laboratory, 7000 East Ave, Livermore, CA 94550 USA; 5 Virginia Tech, Blacksburg, 24061 USA

**Keywords:** Undervaccination, Spatial clustering, Scan statistics

## Abstract

**Background:**

Clusters of under-vaccinated children are emerging in a number of states in the United States due to rising rates of vaccine hesitancy and refusal. As the measles outbreaks in California and other states in 2015 and in Minnesota in 2017 showed, such clusters can pose a significant public health risk. Prior methods have used publicly-available school immunization data for analysis (except for a few, which use private healthcare patient records). School immunization data has limited demographic information—as a result, such analyses are not able to provide demographic characteristics of significant clusters. Further, the resolution of the clusters identified by prior methods is limited since they are typically restricted to disks or well-rounded shapes.

**Methods:**

We use realistic population models for Minnesota (MN) and Washington (WA) state, which provide a model of activities for all individuals in the population. We combine this with school level immunization data for these two states, to estimate vaccine coverage at the level of census block groups. A scan statistic method defined on networks is used for finding significant clusters of under-immunized block groups, without any restrictions on shape. Further we provide the demographic characteristics of these clusters.

**Results:**

We find 2 significant under-vaccinated clusters in MN and 3 in WA. These are very irregular in shape, in contrast to the circular disks reported in prior work, which rely on the SatScan approach. Some of the clusters found by our method are not contained in those computed using SatScan, a state-of-the-art software tool used in similar studies in other states.

**Conclusions:**

The emergence of under-immunized clusters is a growing concern for public health agencies because they can act as reservoirs of infection and increase the risk of infection into the wider population. Higher resolution clusters computed using our network based approach and population models provide new insights on the structure and characteristics of such clusters and enable targeted interventions.

## Background

There have been several outbreaks of vaccine preventable diseases in the US, such as a large measles outbreak in Minnesota in 2017 and a multi-state measles outbreak linked to an amusement park in California in 2015. Such outbreaks have been linked to decreasing vaccination coverage—and while vaccination coverage for the measles, mumps and rubella vaccine (MMR) remains high on average, there are regions with significantly low coverage. For instance, according to the Centers for Disease Control and Prevention (CDC), 95% of children in kindergarten in the US have had 2 doses of MMR vaccine, which is a high enough rate to reach herd immunity. However, several past studies have identified spatial clusters with significantly lower than average vaccination rates for MMR and other vaccines, increasing their susceptibility to outbreaks and creating a public health risk for the communities surrounding these clusters e.g., [1–4].

Identifying the factors associated with under immunized clusters is important because it can help to more accurately identify and characterize populations that may be vulnerable to outbreaks of vaccine-preventable diseases. One of the most comprehensive analysis of such factors associated with under immunized clusters was by Lieu et al. [1]—they use detailed data from Kaiser Permanente of Northern California (KPNC), a managed care provider, to identify various demographic factors such as race and income, which characterize under-immunized MMR clusters in Northern California. This data contains a lot of detail about patients in the KPNC network, but this methodology cannot be easily extended to other states where such data are not available, and one has to rely on school level immunization data (e.g., as used in [4]), which is often the only data available. Additionally, the patient data used in [1] only covers less than half the population, which might lead to certain biases.

Spatial scan statistics provides a rigorous method for identifying statistically significant clusters [5], and have been used in many past studies, such as [1, 4, 6]. The standard use of this approach (using the SatScan software) involves scanning the entire region using a circular shaped window (which is a spatial window, defined using Euclidean distance) to find a cluster that optimizes a maximum likelihood objective; this has been extended to other shapes, such as ellipses. However, because of the restriction in shapes, this approach does not find high resolution irregularly shaped regions—if “real” clusters do not have the prescribed shape, SatScan will miss them or only partially catch them. We note that Delamater et al. [7] developed a methodology to combine school immunization data with a gravity based population mobility model; however, they do not use scan statistics for identifying significant clusters.

Our goal is to use publicly available school level immunization data to identify high resolution under immunized clusters (see [8] for more details on the availability of such data). Our specific objectives are: (1) develop a methodology to integrate school level immunization data with detailed population models to construct a block group level immunization rate model, and (2) identify high resolution statistically significant clusters, which may be irregularly shaped, and give new insights into the regions of concern beyond what SatScan can provide.

## Methods

We construct an immunization rate model at the census block group resolution. The steps involve integrating activity-based populations for Minnesota (MN) and Washington (WA) with school immunization data collected by the health departments in these states.

### Activity-based model and demographics

Following the approach of [9, 10], we use a population model that represents the entire population of MN and WA, with complete demographics and activities for each person, activity times, and locations. This representation integrates over one dozen public and commercial datasets. We briefly summarize the steps of the process here and refer to [9, 10] for complete details. As a first step, a synthetic representation of each person in the population is constructed who, when aggregated to a block group level, are statistically equivalent to individuals in the U.S. census block group.

Next, daily activities are assigned to individuals within a household using activity and time-use surveys, and methods from transportation science. Finally, activity locations are determined using methods from transportation studies and detailed land use data. In particular, this model has school locations for all school aged children. The resulting population—referred to as an “activity-based population”— is statistically equivalent to the census; we refer to [9, 11–14] for details on validation. The population models developed using this approach have been used in a number of studies on epidemic spread and public health policy planning [9, 10, 12, 15–18].

Here, we use activity-based models constructed using the 2010 census data. These models have rich demographic characteristics; in our study, we focus on age and income. We divide the population by age into four categories: Pre-school (ages 0–5), school (ages 6–17), adults (ages 18–65), and senior (ages 65+). These groups represent 7, 19, 67, and 7% of the population, respectively. We also group the population by annual household income into low ($0 —$25, 000), medium ($25 000 —$75,000), and high ($75,000+), which represent 20, 45, 35% of the population, respectively. We use the same categories for the state of Washington.

### School immunization data

We describe here the publicly available school immunization data that we use in our analysis for MN and WA. In this paper, we only focus on the immunization rates for the MMR vaccine for middle school children, namely, 7th grade in the case of Minnesota and 6th grade in the case of Washington, since any children who get delayed immunizations (i.e., those do not get the school-entry vaccine requirements by elementary school, but do so in later years), are likely to be covered in this data.

#### Minnesota

We use data collected by the Minnesota Department of Health (MDH) for the school year 2015–2016, as reported in their Annual Immunization Status Report (AISR).^1^ The data contains immunization statistics for 7th-grade students in all public and private schools across the state, except for schools with fewer than 5 children—these are not available because of privacy—and schools that did not respond to MDH. Initially, there are 872 schools in the MDH report, and after removing schools with fewer than 5 children (114) and schools that did not report (12), we are left with 712 schools. The relevant fields for this study are 1) total enrollment and 2) percentage of students who have received two doses of the MMR vaccine. From this data, we compute the number of unvaccinated and under-vaccinated^2^ children in a school as

$$ \left(\mathrm{total}\kern0.5em \mathrm{enrollment}\right)\kern0.5em \times \left(1-\frac{\%\mathrm{vaccinated}}{100}\right) $$

We use the vaccination rates from the AISR to assign an immunization status to each children in the activity-based model. In order to perform this assignment (Section 2.3), we need to find a mapping between schools in the MDH data and synthetic schools in our population model. However, the data reported by MDH only lists school names and their corresponding school districts. We need a full address for each school, in addition to the name, in order to find the corresponding school in the activity-based model. Addresses were obtained from the Minnesota Department of Education website or, when not available on the website, by manual search using Google Maps.

#### Washington

Immunization data for Washington was obtained from the Washington State Department of Health (WDH) website.^3^ The report contains entries for 6th-grade students in 2615 public and private schools across the state, for the school year 2016–2017. However, a majority of the schools did not have enrollment data (137) or had a reported enrollment of zero students (1441). We discarded these entries as well as all the schools with less than 5 students (138) to obtain a total of 899 schools. The WDH data does include full addresses for each school, allowing us to skip one step in data collection and manual labeling. Then, the immunization data is used to model immunization rates, as described in the next subsection.

### Modeling immunization rates in the activity-based population

The objective here is to estimate the immunization rate for MMR for each census block group. We note that the school immunization data does not immediately give us these estimates, since a school serves children from many block groups, typically the closest ones. We only consider children who are not home schooled. Our method involves the following steps.

**Fig. 1:**
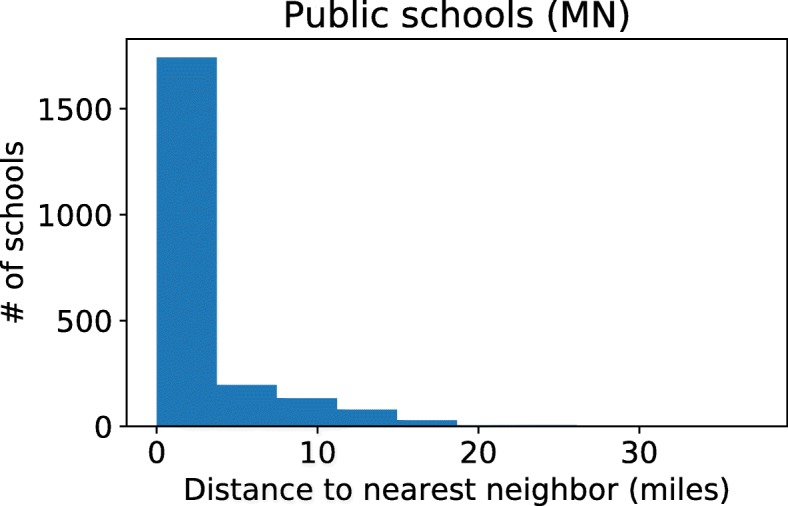
Distribution of the matching distances in MN

1. After obtaining addresses for all the schools, we perform an inexact string matching based on the Levenshtein distance [19] to find pairs of schools in the activity-based model and the MDH data. We were able to find 641 schools in the model that matched to one of the 712 schools in the MDH report, both in name and in location, as verified using the addresses. Let A denote the set of schools in the activity-based model, and let *B* ⊆ *A* be those whose name matched with some school in the MDH data.
2. The activity-based model has many more schools, 3107 in total. After the initial string-based assignment, we match the remaining schools (i.e., those in *A*−*B*) to the geographically closest school in the set B, for which we found a string match. For example, for the school “BLUE EARTH AREA SR.” in the MDH data, we find a school with the same name in the activity-based model (string match). We did not find a string match for the synthetic school “BLUE EARTH AREA ALP”, so this school gets the same vaccination rate as the nearest neighboring school in the model, which happens to be “BLUE EARTH AREA SR.” Figure 1 shows a box plot of the distances from schools in A to the corresponding mapped one in the MDH data.
3. The activity-based population has an assignment of each child v to a school S(v), which also includes home schools. For each child v, who is not home-schooled, we estimate a probability (denoted by p(v)) of getting both doses of MMR to be the immunization rate in the school S(v).
4. Finally, we aggregate the population by census block group. The expected number of immunized children in block group b is Σ_*v∈C*(*b*)_ *p*(*v*), where C(b) is the set of children with home address in b. The immunization rate in the block group b is the average probability of children in b being vaccinated, i.e., Σ_*v∈C*(*b*)_ *p*(*v*)/|*C*(*b*)|. The statewide vaccination rate is 96.2%.
  In performing the distance-based matching, we impose a rule that a public school cannot be matched to a private school and vice versa, with the goal of accounting for differences in demographic properties and immunization practices in public and private schools. We do highlight that the distance-based matching is a somewhat crude approximation in the absence of other data, with the potential for losing demographic information, and this is a limitation of the study.
  We follow the same process for the WDH data. There are 2,813 schools in the activity-based model, and we were able to find a string-match for 571 of those to some school in the WDH report. Distance-based matching was used to assign immunization rates to the remaining schools. We obtained a statewide vaccination rate of 95.7%. Table 1 summarizes the number of schools in the MDH and WDH reports, the number of schools in the activity-based model, and the matches to obtain an immunization rate for each school. We note that there is a significant mismatch in the number of schools in the activity based population and the data from MDH and WDH. This is likely to be a combination of multiple reasons, including: (1) Potentially missing schools in the data from MDH and WDH: for instance, schools with schools with fewer than five children, and home school information is missing; (2) The population model is based on 2010 census data (as discussed in Section 4.2), which might have had more schools, and (3) It is possible that some of the schools in the activity based population are not middle schools (home schooled children are included in the model).

**Table 1:** Number of schools in the immunization data and the activity-based model

State	School type	Schools in immunization data	Schools in activity based model	String matches	Distance matches
Minnesota	Public	433	2588	390	2198
	Private	279	519	131	388
Washington	Public	682	2245	509	1736
	Private	217	568	62	506

### Statistical analysis using network scan statistics

We use the methods of scan statistics [5, 20, 21] to identify statistically significant geographical clusters with a high proportion of under-immunized children—this approach formalizes anomaly detection as a hypothesis testing problem, and has been used for detecting anomalies or “hotspots” in spatial data [22–24]. We use an extension of this approach to networks, which has been used for anomaly detection in network data [25–27]. Specifically, we consider a network G = (B, E) defined on the set of block groups, i.e., each block group b is a node in the set B.

Two block groups *b*, *b*′ ∈ *B* are connected by an edge, (*b*, *b*′) ∈ *E*, if they share a boundary. Thus, G denotes the adjacency graph of the block groups. We say that a subset *C* ⊂ *B* is a connected subgraph if the graph *H*(*C*, *E*′) formed by considering only the edges (*b*, *b*′) ∈ *E* with *b*, *b*′ ∈ *C* is connected. We consider such subgraphs, since this allows us to consider clusters of arbitrary shapes, whereas most applications of scan statistics in spatial data restrict the clusters to have some fixed regular shape. We note that a cluster of block groups that is topologically shaped as a circle is also a connected subgraph, with respect to the above definition. Therefore, this notion strictly generalizes the clusters considered in SatScan.

For each block group *b* ∈ *B*, we have two counts: (i) pop(b), which is the baseline count of 7th grade children in MN (6th grade in WA), and (ii) unimm(b), which is the estimated number of under-immunized 7th grade in MN (6th grade in WA) children (also referred to as the “event count”). Following our notation defined earlier, we have *unimm*(*b*) = Σ_*v∈ C*(*b*)_(1− *p*(*v*)). We use the Poisson version of the Kulldorff scan statistic [5], where the null hypothesis *H*_0_ is that the event counts for all nodes b are generated proportionally to their baseline counts, i.e., (1− *μ*) ∙ *pop*(*b*), where *μ* is the state-wide immunization rate.

Under the alternative hypothesis H_1_(C) for a cluster C, the event counts for nodes outside C, i.e., in *B*−*C* are (again) generated with rate proportional to the baseline counts, but for the nodes within C, the counts are generated at a higher rate than expected. The scan statistic F (C) is defined as a generalized log-likelihood ratio, and the objective is to find clusters that maximize this statistic. In the classical spatial scan approach, which is implemented in the popular SatScan software [28], the maximization is done over circular and elliptical regions.

Optimization over clusters of arbitrary shape is computationally much more challenging. We will use the approach of [29], which efficiently searches over all connected clusters of a certain size and provably finds one with the maximum log-likelihood score. The restriction on the cluster size serves as a regularization constraint, while also making the problem computationally more tractable. We note that there are many other potential approaches for finding such clusters, e.g., greedily picking significant block groups, and connecting them subsequently. However, it is shown in [29] that approaches such as this do not perform very well, in general. Monte Carlo sampling is used to determine the p-value for each cluster—accounting for multiple hypothesis testing— and we consider the top few significant clusters. We compare our results with the clusters discovered using SatScan.

Many extensions of scan statistics allowing arbitrary shapes have been proposed, e.g., [30, 31]. We also note that there is some risk of finding spurious or very “patchy” clusters, when constraints on the allowed shapes are relaxed. In particular, an “octopus” effect has been reported [30], where clusters with high even counts are connected by narrow paths on a network. We explore this possibility through simulations in the Appendix. We find that if the target cluster is very different from the background population, the maximum log-likelihood cluster has a high overlap with the target. On the other hand, if the true cluster is not very significant, the maximum log-likelihood solutions might differ quite a bit. This is related to, but not as extreme as the octopus effect. We hypothesize that the constraint on the solution size and the optimality guarantee in the method of [29] prevents reaching the degenerate cases reported in [30]. For a more detailed discussion of the advantages and limitations of scan statistics, we refer to [5, 27, 31].

### Characterization of under-immunized block groups

To characterize the block groups that are a part of the anomalous clusters of under-immunized children, we perform separate logistic regression analysis for MN and WA using all the block groups in each state. The response variable in the regression is whether the block group is a part of the under-immunized clusters or not. It takes value 1 if it is a part of one of the under-immunized clusters and 0 if it is not. The independent variables we considered are average age in the block group, number of workers per capita, average household income in block group, average household size, number of children in age group 0-5 years and the total income of the block group. These variables were selected because data for them are available as part of the census data.

The raw feature list considered other demographics as well such as the number of school aged children 6-18 years, number of adults between 19-65 years, number of people older than 65 years, and the total population size of the block group. However these variables were correlated with the number of children 0-5 years, and hence were removed to avoid the problem of multi-collinearity.

The regression analysis identifies the list of statistically significant demographics that contribute to the probability of a block group being a part of the under-immunized cluster. If we can identify these features in a robust manner, public health officials can utilize this information to design actionable and targeted policies.

## Results

We discuss our results for under immunized MMR clusters, and we describe the size, location, and demographic characteristics of the clusters. We consider the clusters at a statistical significance level of p < 0.05, and compare with those found using SatScan. Finally, we use a regression analysis to explain the block groups involved in the significant clusters.

### Under-vaccinated clusters: Size and location

#### Minnesota

We discover 2 significant under-vaccinated clusters in Minnesota. In Table 2, we report the size of the clusters in terms of number of block groups, total children, and unvaccinated children. In the first cluster, the vaccination rate is only 81.2%; in contrast, recall that the statewide average is over 96.2%. The second cluster has above 90% vaccination rate, but it affects a larger population and a larger geographical region. The third cluster is marginally significant but has an alarmingly low vaccination rate of only 72.9%—although it affects a smaller set of children.

**Table 2:** Under-vaccinated clusters in Minnesota

# Block Groups	# Children	# Unvaccinated (Expected)	Vac. Rate (%)	p
187	3567	668 (136.84)	81.2	0.001
167	4301	390 (165.00)	90.9	0.018
16	233	63 (8.93)	72.9	0.052

**Fig. 2:**
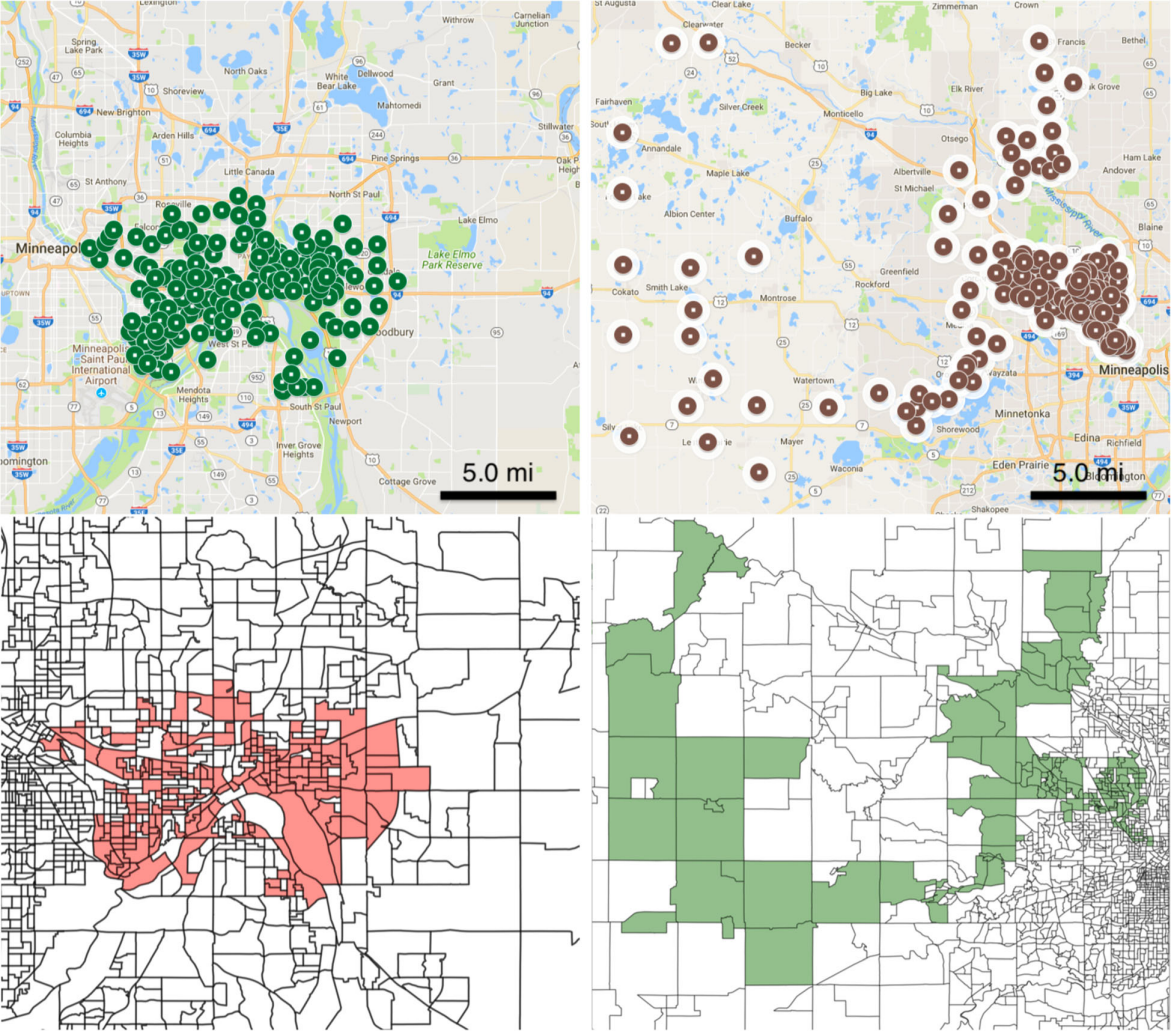
Top two significant clusters in MN (top right and top left) are shown. Each dot on the map is a block group. The same clusters are shown as block group polygons on the bottom right and left, with each marker corresponding to a block group. First cluster in Minnesota covers the city of St. Paul (top left and bottom left) and the second cluster covers the rural block groups west of Minneapolis (top right and bottom right)

In Fig. 2, we show the two significant clusters over a map of MN with each marker corresponding to a block group (top) and as polygons (bottom). We notice some differences between both clusters. The most significant cluster—with vaccination rate 81%—covers the Twin Cities of of St. Paul and Minneapolis—a dense, urban region—and it is compact in shape. The second cluster, on the other hand, spans a more rural area west of Minneapolis, and it extends over a much larger geographical region. In the next subsection, we analyze, the demographic properties of these two clusters.

#### Washington

For Washington state, we discover 3 significant clusters (Table 3). The vaccination rate in these regions varies from 78.2% to 85.4%; in contrast, the statewide rate is 95.7%. The number of unvaccinated children is much lower than in the Minnesota clusters.

**Table 3:** Under-vaccinated clusters in Washington state

# Block Groups	# Children	# Unvaccinated (Expected)	Vac. Rate (%)	p
51	974	179 (40.9)	81.6	0.001
44	500	109 (21.0)	78.2	0.012
51	1048	153 (44.0)	85.4	0.042

**Fig. 3:**
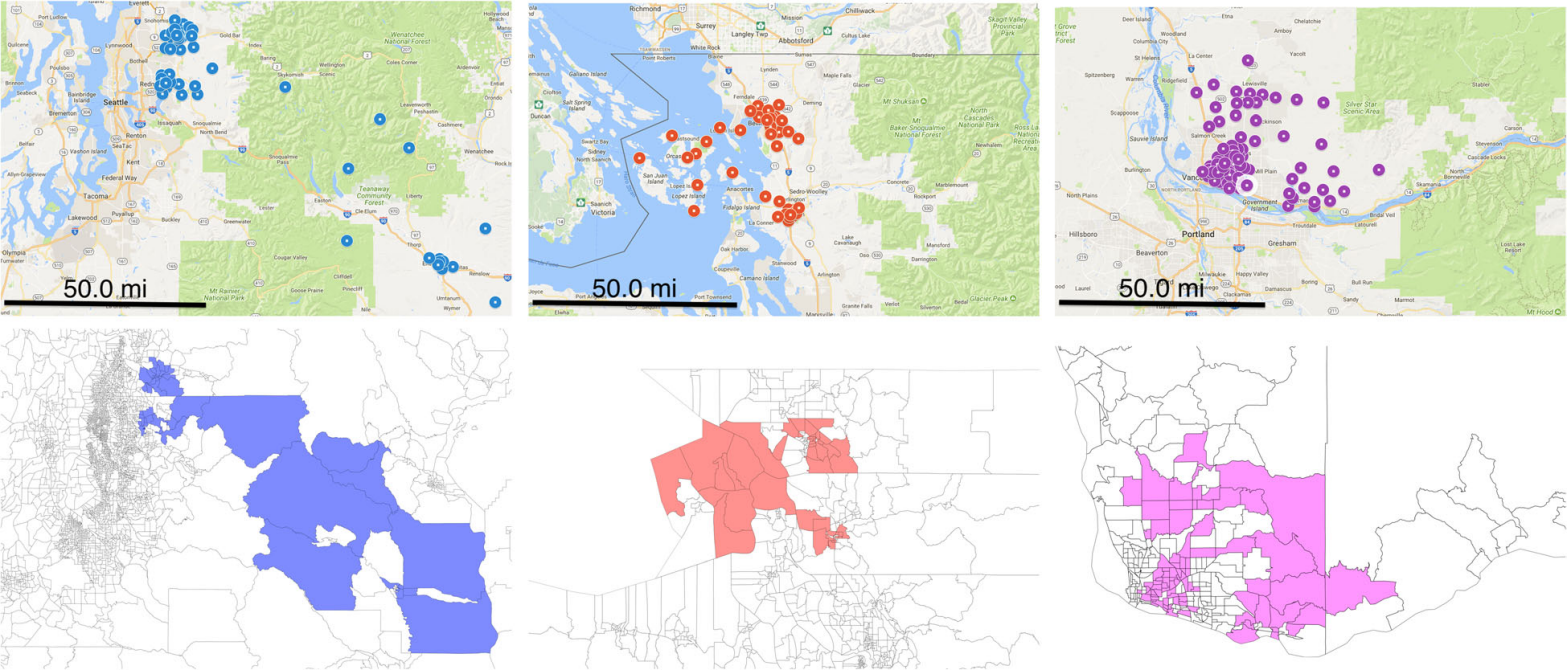
Undervaccinated clusters in Washington state are centered in major cities. Each subfigure on the top corresponds to a cluster. The bottom parts of the plot are showing the same clusters drawn as polygons

When drawn over a map of Washington (Fig. 3), we see that the significant clusters span different parts of the state instead of being concentrated on a single area. In particular, the clusters are associated with major metropolitan areas—Seattle, Bellingham, and Vancouver—and then span out to rural regions.

### Demographic properties

We compare the distributions of demographic properties within the significant clusters with the corresponding statewide distributions.

#### Minnesota

**Fig. 4:**
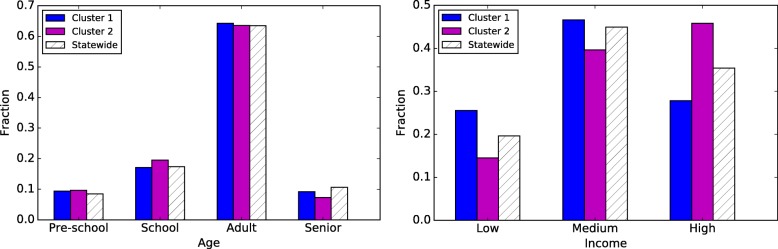
The age (left) and income (right) distributions for the significant clusters and the entire state of Minnesota

We observe notable differences between the most significant cluster and the entire state in terms of income distribution (Fig. 4 (right)). In the cluster, the percentage of low-income households is 6 points higher than the statewide distribution, whereas there is a decrease of 8 percentage points in the high-income population. The over-representation of low-income households is interesting, considering that this cluster spans a major metropolitan area. On the other hand, the age distribution in the cluster does not deviate from the statewide average.

In the second cluster, we find that the high-income households are over-represented by 11 percentage points, with an even decrease on low and medium income households, though this difference may be attributed to the location of the cluster—mostly over Minneapolis. Again, we do not observe differences in the age distribution.

#### Washington

**Fig. 5:**
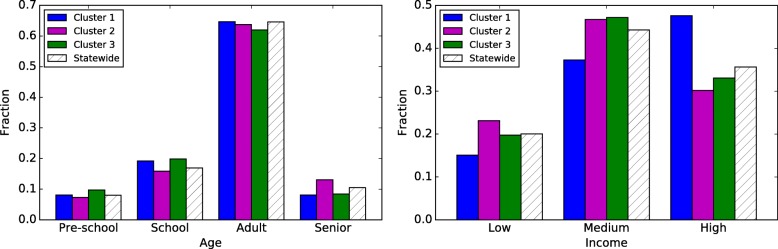
The age (left) and income (right) distributions for the significant clusters and the entire state of Washington

Our observations are similar in the three clusters discovered in Washington state (Fig. 5). The most significant cluster has an over-representation of high-income households, while the second cluster has more low-income households than in the statewide distribution. High-income households are over-represented by roughly ten percentage points in the most significant cluster and low-income households are overrepresented by four points in the second most significant cluster. This is not surprising as the former cluster is, for the most part, concentrated in the suburbs around Seattle. There is a slight increase in school-age population in the Seattle and Vancouver clusters.

### Comparison to SatScan

**Fig. 6:**
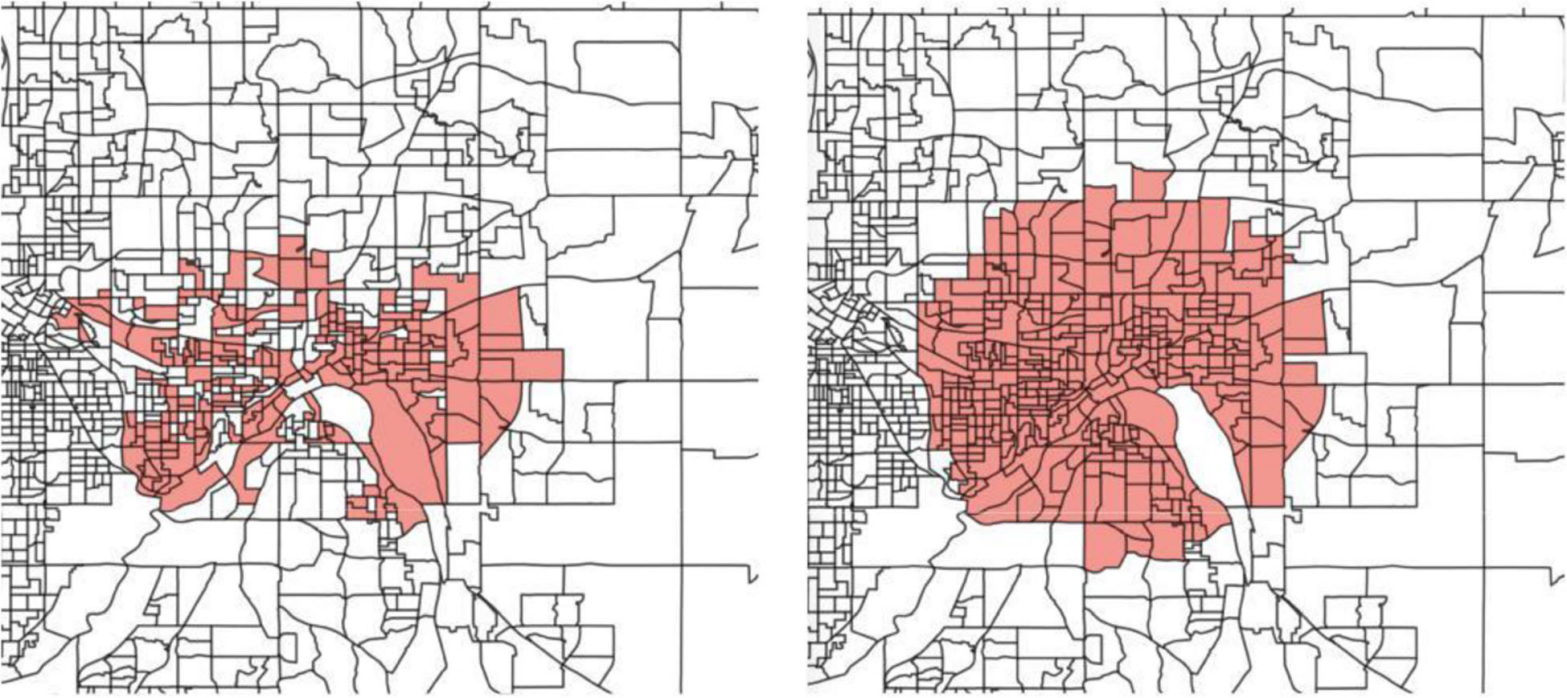
Most significant cluster in Minnesota using network scan statistics (left) compared to SatScan (right). By searching for arbitrary shapes, we capture a cluster with lower vaccination and higher statistical significance

In Fig. 6, we show the most significant cluster in Minnesota discovered using the network-based scan statistics (left), as well as the most significant cluster discovered using SatScan (right). Both clusters cover the same area of Minnesota—the Twin Cities—however, we obtain a better fit of the under-vaccinated region. The SatScan cluster has a likelihood ratio score of 436.27, compared to 577.80 in the one that we discover—i.e., our cluster is more significant. Furthermore, the vaccination rate in the left cluster is lower, 81% compared to 88%.

**Fig. 7:**
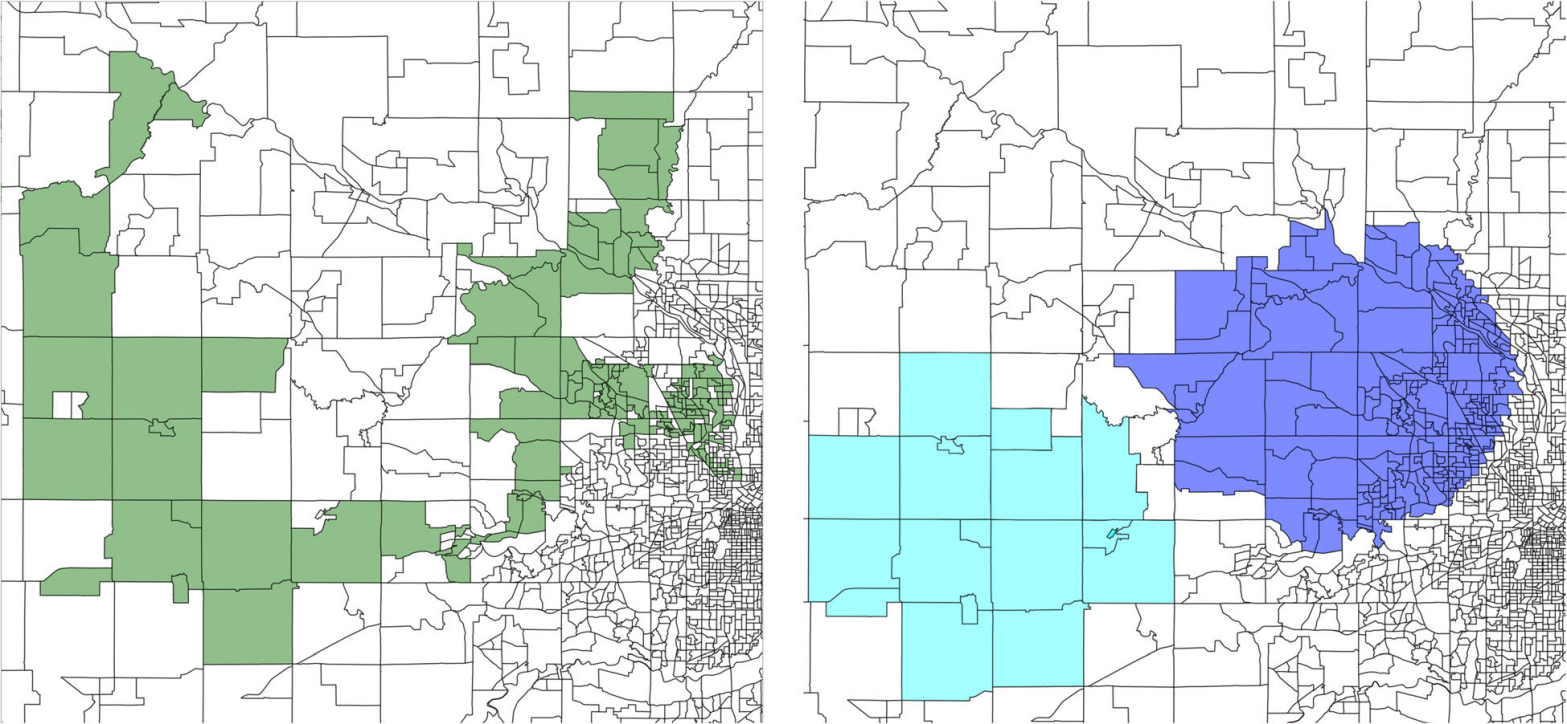
Second significant cluster in Minnesota using network scan statistics (left) and SatScan (right). SatScan has to approximate irregularly-shaped regions with circles

We also find that SatScan has difficulty detecting regions of irregular shape, such as the “U”-shaped cluster in Minnesota (Fig. 7). Using SatScan, this cluster is approximated by two smaller circular regions; this hinders the ability of public health officials to allocate resources efficiently throughout the cluster and to interpret the results of the analysis.

Another weakness of only looking for regions of a predetermined shape—i.e., circles—is the potential to overestimate the statistical significance of the clusters. Recall that statistical significance of the scan statistic is assessed via Monte Carlo simulation. This simulation gives us the minimum likelihood ratio score that a cluster should have to be considered significant at some level—0.05, 0.01, etc. However, this threshold is much lower if we make the prior assumption that the under-vaccinated clusters are circular.

In Table 4, we show the likelihood ratio scores of the 6 clusters that SatScan detected as significant with p < 0.05. Specifically, these clusters were flagged because they have scores above 11.11, which was the threshold for significance established through Monte Carlo simulation. Only four of these clusters would be significant at p < 0.01. On the two rightmost columns of the table we report the threshold for significance established with network scan statistics.

**Table 4:** Statistical significance of circular clusters is overestimated

		Likelihood Ratio Threshold for Significance			
Top clusters	Likelihood	SatScan 0.05	SatScan 0.01	ColCodeNP 0.05	ColCodeNP 0.01
		11.11	13.00	69.89	142.02
1	436.27	✓	✓	✓	✓
2	69.20	✓	✓	✗	✗
3	27.16	✓	✓	✗	✗
4	14.59	✓	✓	✗	✗
5	12.65	✓	✗	✗	✗
6	11.62	✓	✗	✗	✗

We note that this threshold is much higher, and only the top cluster discovered by SatScan (Fig. 6) would be declared significant at either the 0.05 or 0.01 level. To summarize, it is perfectly reasonable to look for regions of a fixed shape if we have some prior knowledge about what the clusters of interest look like. However, if in reality there are under-vaccinated regions of arbitrary shape, we will be prone to overestimating the significance of the predetermined shapes.

**Fig. 8:**
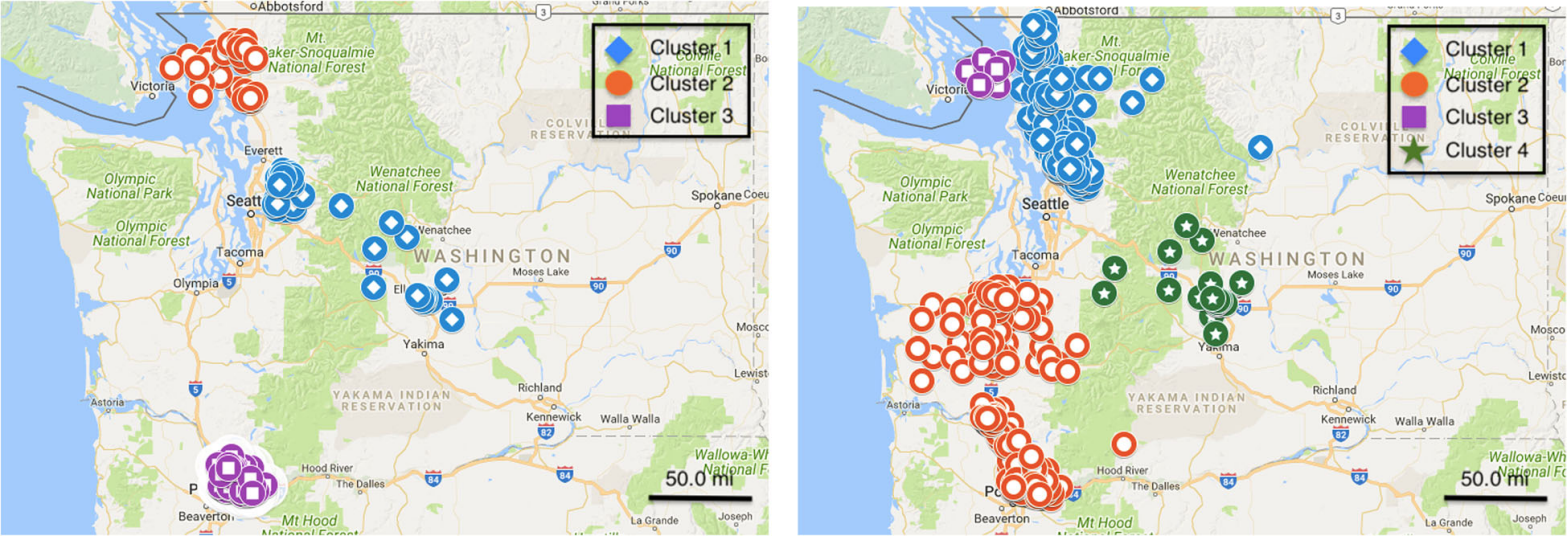
Significant under-vaccinated clusters discovered using network scan statis-tics (left) and SatScan (right) in Washington state. Blue diamond markers correspond to the most significant cluster, followed by red circles, purple squares, and green stars

Similarly, we observe differences in the significance assignment from both methods in Washington (Fig. 8). The most significant region we find spans western Red-mond, whereas SatScan declares the Bellingham cluster to be the most significant. In fact, SatScan misses the Redmond block groups completely.

### Characteristics of the under-immunized block groups

Recall that we perform a logistic regression to predict whether a block group is part of the under-immunized clusters or not (the response variable). We report the results in Table 5. In Minnesota, all demographics except the total income of the block group are statistically significant in explaining the likelihood of a block group being a part of an anomalous under-immunized cluster. However, the magnitude of the impact from household income and the number of children between ages 0-5 years is almost negligible. Average age in the block group, number of workers per capita, and the average size of the household are all negatively correlated with the response variable.

**Table 5:** Logistic regression analysis. The response variable is whether the block group is a part of the under-immunized clusters or not. The independent variables are average age in the block group, number of workers per capita, average household income, average household size, number of children in age group 0–5 years and the total income of the block group

Variables	Minnesota		Washington	
	Coefficient	p-value	Coefficient	p-value
avg. age	−0.161	<: 001 ^***^	−0.029	0.026 ^*^
workers per capita	−7.28	<: 001 ^***^	−4.45	<: 001 ^***^
avg. household income	1.088e-05	0.0012 ^**^	−7.416e-06	0.08 ^*^
avg. household size	−0.91	<: 001 ^***^	0.167	0.32
# of children 0–5 yrs.	−3.742e-03	0.012 ^*^	−5.762e-04	0.62
total income	5.911e-09	0.19	1.804e-08	<: 001 ^***^

In Washington, age, workers per capita, household income, and total income in the block group are all statistically significant in explaining the response variable. Age, workers per capita, and household income are negatively correlated whereas total income is positively correlated to the response variable. Again, the impact of household income and the total income on the outcome is almost negligible.

The two variables that are consistently significant and influential in both MN and WA are the average age in the block group and the number of workers per capita. Both have a negative influence on the response variable. A possible explanation for the negative relationship with average age is that, as the average age in block group increases, there are fewer children left to be labeled under-immunized, which reduces its chances of being in an anomalous cluster. In the case of the number of workers per capita, a higher number of workers may imply a higher level of education in the block group, which may imply a higher likelihood of vaccinating children in workers’ families.

## Discussion

### Major findings

First, we discuss the structure of the clusters we find. Our results indicate existence of block group clusters with significant under-immunization rates for MMR, both in Minnesota and Washington. These clusters are generally small, have irregular shape, and they are not identified by the SatScan method—although they overlap with the clusters identified by SatScan. Additionally, the clusters we report have higher likelihood score and generally lower immunization rates than those reported by SatScan, which signals a higher potential threat from these clusters. Further, by imposing a prior assumption that clusters are circular, SatScan over-estimates the statistical significance of circular regions. Thus, our results demonstrate the additional insights that can be obtained by considering irregularly-shaped clusters using scan statistics on a network representation. In particular, if “real” clusters do not have the prescribed shape, which is likely to be the case, SatScan will miss them or only partially identify them. The higher resolution clusters can also make it easier to intervene in a targeted manner, and allocate public health resources more optimally.

Next, we consider the demographic characteristics of the populations within the clusters we find. The age distributions in the top two clusters in MN and the top three clusters in WA do not show any notable differences from the statewide distributions. However, they show significant differences with respect to income distributions, but these are not consistent among the clusters. Note that these observations are about the population within the individual clusters.

Our regression analysis considers a different quantity, namely, whether a specific block group is contained in any of the significant under immunized clusters. Our analysis shows that two variables, namely, the average age and the number of workers per capita in a block group, are predictors for the block group to be contained in a significantly under-immunized cluster. The fact that age is a significant factor might seem inconsistent with the cluster-level observation stated above. We emphasize that the regression analysis is considering a different quantity from the within cluster distributions, so this is certainly plausible. However, more research is needed to understand the robustness of these observations, and the likely causes. Finally, we note that the school immunization data by itself does not have rich demographic information to enable such analyses. The first component of our methodology, namely the integration of an activity based detailed population model with school immunization data gives an individual level immunization model, which enables such an analysis.

### Limitations

Our study is focused on 6th and 7th grade children. The results might be different when we consider other age groups, and would be an interesting research question as well. There are potentially several sources of missing data and inconsistencies and uncertainty in the datasets we use. Uncertainties in the activity based population model have been identified in earlier studies on infectious diseases [9, 10, 12]. The population matches census at a block group level, so we expect the spatial resolution we have considered will limit the impact. The biggest source of inconsistency is that the school immunization data is for 2015-2016, whereas the activity based population is based on the 2010 census data. There is also an inconsistency between the number of schools listed in the MDH and WDH datasets, and the population data. There might be multiple reasons at play here, as discussed below (sources of uncertainty), and in Section 2.3. The methodology we develop here can be applied without any changes once an updated activity based population is constructed. We note that other agent based populations have been used for studying measles outbreaks [32], but the specific methodology is different from [9, 10, 12], which is the basis of our work.

Some of the sources of uncertainty with respect to the school immunization data are: (1) data for schools with fewer than five children is not available, (2) there is no data on home schooled children, and (3) charter schools are likely being overrepresented by our matching method. Charter schools generally have little to no nursing staff and lack technological resources, which impacts their immunization data quality.

## Conclusions

The emergence of under immunized clusters is a growing concern for public health agencies. Higher resolution clusters computed using our network based approach can provide additional insights on the structure and characteristics of such clusters. The integration of an activity based population with publicly available data, such as school immunization records, can potentially be useful in other analyses. We note that our analysis has been done with the publicly available school immunization data, and does not reflect any official conclusions of the public health departments of MN or WA.

## Figures and Tables

**Fig. 9:**
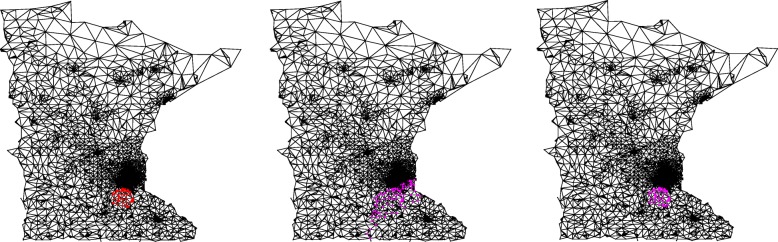
Planted circular under-vaccination cluster in Minnesota (left) and discovered clusters using network scan statistics when the number of under-vaccinated children inside the cluster is twice (center) and 10 times (right) larger than the statewide rate

**Fig. 10:**
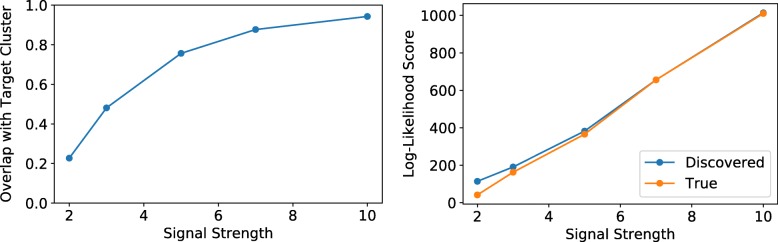
Left: Overlap between the discovered cluster and the target circular cluster for different signal strength levels. At low signal strength, the network scan statistic approach finds cluster with poor overlap with the target. Right: Log-likelihood scores of the true and discovered clusters. At low signal strength, the larger regions found using network scan statistics also have notably higher scores than the real region

## Appendix

**Sensitivity of Network Scan Statistics for Circular Clusters**

By allowing the network scan statistics method to scan over regions of arbitrary shape, it is possible to discover elongated and “U”-shaped clusters that would be misrepresented if we were to constrain the shape. However, we note that when the target cluster is in fact circular, the network scan statistic approach discovers larger regions centered around the target. This behavior has been documented before in a comparative study of algorithms for scan statistics optimization [31].

As an example, we inject a circular cluster in the block group network of Minnesota. Figure 9 (left) shows the block groups in the target cluster in red. For this experiment, we set the statewide unvaccination rate at α=0.038. Inside the cluster, we use multiples of the statewide vaccination rate: 2α , 3α , 5α , 7α , 10α. We refer to the multiplying factor as the signal strength. In all cases, the network scan statistics approach finds a cluster that overlaps with the target. However, for a signal strength of 2 and 3, the discovered cluster has about twice the size of the target. For a signal strength of 5 and above, we observe high overlap with the target. These observations are summarized in Fig. 10 (left), where we show the overlap—size of intersection divided by size of union—between the target and discovered regions. Further investigating this discrepancy, we see that with the network approach, we always discover clusters with a higher log-likelihood score—i.e., more evidence of being anomalous—than the target cluster. As shown in Fig. 10 (right), the discrepancy is more noticeable at the lower signal strength levels, which correlates with the low overlap at these levels.

In summary, using network scan statistics, we are able to recover regions with higher log-likelihood scores than what is obtained by constraining the search to circular shapes only. Finding higher scores is a desirable property, since these scores translate to more support for the alternative hypothesis of an anomalous cluster (Section 2.4). However, as we saw above, score optimization by itself may lead to spurious results, which is a limitation the scan statistics methodology in general. In practice, if there is a good reason to believe that the target anomaly is of a given shape—i.e., a circle or a square—the best way to proceed is to optimize the likelihood score and conduct a significance test on clusters of this shape only, as observed in [31]. When a given shape is not a reasonable assumption, but there is knowledge about the expected size or radius of the anomaly, network scan statistics with a size constraint would be a better alternative. Finally, based on the results from Fig. 10, when using network scan statistics, one may choose to be skeptic of discovered clusters with rates lower than 3 times the rate of the entire region under study.

## Footnotes

1
http://www.health.state.mn.us/divs/idepc/immunize/stats/school/

2 We do not distinguish between children who have received one dose and children who have received no dose.

3
https://www.doh.wa.gov/DataandStatisticalReports/HealthBehaviors/Immunization/SchoolReports/DataTables
